# Spinal versus general anaesthesia in surgery for inguinodynia (SPINASIA trial): study protocol for a randomised controlled trial

**DOI:** 10.1186/s13063-016-1746-x

**Published:** 2017-01-14

**Authors:** Willem A. R. Zwaans, Léon H. P. M. le Mair, Marc R. M. Scheltinga, Rudi M. H. Roumen

**Affiliations:** 1 Department of General Surgery, Máxima Medical Centre, PO Box 7777, 5500 MB Veldhoven/Eindhoven, The Netherlands; 2 SolviMáx, Center of Excellence for Abdominal Wall and Groin Pain, Máxima Medical Centre, Eindhoven, The Netherlands; 3 Department of Anaesthesiology, Máxima Medical Centre, Veldhoven, The Netherlands

**Keywords:** Anesthesia, Pain, Postoperative, Randomised controlled trail [publication type], Chronic pain, Pain management, Surgical procedures, Operative

## Abstract

**Background:**

Chronic inguinodynia (groin pain) is a common complication following open inguinal hernia repair or a Pfannenstiel incision but may also be experienced after other types of (groin) surgery. If conservative treatments are to no avail, tailored remedial surgery, including a neurectomy and/or a (partial) meshectomy, may be considered. Retrospective studies in patients with chronic inguinodynia suggested that spinal anaesthesia is superior compared to general anaesthesia in terms of pain relief following remedial operations. This randomised controlled trial is designed to study the effect of type of anaesthesia (spinal or general) on pain relief following remedial surgery for inguinodynia.

**Methods:**

A total of 190 adult patients who suffer from unacceptable chronic (more than 3 months) inguinodynia, as subjectively judged by the patients themselves, are included. Only patients scheduled to undergo a neurectomy and/or a meshectomy by an open approach are considered for inclusion and randomised to spinal or general anaesthesia. Patients are excluded if pain is attributable to abdominal causes or if any contraindications for either type of anaesthesia are present. Primary outcome is effect of type of anaesthesia on pain relief. Secondary outcomes include patient satisfaction, quality of life, use of analgesics and (in)direct medical costs. Patient follow-up period is one year.

**Discussion:**

The first patient was included in January 2016. The expected trial deadline is December 2019. Potential effects are deemed related to the entire setting of type of anaesthesia. Since any setting is multifactorial, all of these factors may influence the outcome measures.

This is the first large randomised controlled trial comparing the two most frequently used anaesthetic techniques in remedial surgery for groin pain. There is a definite need for evidence-based strategies to optimise results of these types of surgery. Besides pain relief, other important patient-related outcome measures are assessed to include patient’s perspectives on outcome.

**Trial registration:**

The protocol (protocol number NL54115.015.15) is approved by the Medical Ethics Committee of Máxima Medical Centre, Veldhoven, The Netherlands. The study protocol was registered at www.trialregister.nl (NTR registration number: 5586) on 15 January 2016.

**Electronic supplementary material:**

The online version of this article (doi:10.1186/s13063-016-1746-x) contains supplementary material, which is available to authorized users.

## Background

### Background and objectives

Chronic postoperative groin pain (inguinodynia) is defined as pain beyond 3 months after inguinal surgery. In the majority of patients, inguinodynia develops following open inguinal hernia repair (66%; Zwaans WA, van Kuijk SM, le Mair LH, van Kleef M, Scheltinga MR, Roumen RM – unpublished observations). One third of patients with chronic postherniorrhaphy pain experience impairment of daily activities [1, 2]. This pain is either neuropathic (47–70%), nociceptive (26%) or a combination [3]. Inguinodynia may also be found after other types of groin surgery [4–7]. Concise history taking and an extensive physical examination provide the cornerstones for diagnosing inguinodynia. A diagnostic local nerve block may confirm the diagnosis [8, 9]. Interestingly, one of three patients benefits from these injections on the long term [10]. When these minimally invasive regimens fail, surgical options may be considered. Removal of the inguinal nerves, funiculolysis, and/or removal of the mesh may all be effective [7, 11–14, 17]. Furthermore, the post-Pfannenstiel pain syndrome, which often is neuropathic in origin, also responds well to an inguinal neurectomy [4].

The SolviMáx Centre of Excellence is a third-line referral centre for abdominal wall and groin pain. Experience with a spectrum of surgical procedures for inguinodynia is growing. One retrospective study in patients with inguinodynia suggested that spinal anaesthesia is associated with a superior outcome in terms of pain relief when compared to general anaesthesia [15]. A more robust trial is required to confirm these preliminary findings. The objective of the present paper is to present a randomised controlled trial that is designed to investigate the effect of type of anaesthesia on long-term pain relief in patients who are surgically treated for chronic inguinodynia.

## Methods

### Trial design

This monocenter, nonblinded, randomised controlled trial is performed in the SolviMáx Centre of Excellence for Abdominal Wall and Groin Pain. SolviMáx is a subdivision of the Surgical Department of Máxima Medical Center (MMC), a teaching hospital situated in the southern part of The Netherlands serving a population of approximately 200,000 inhabitants. The study aims at investigating a potential difference in pain relief following remedial surgery that is performed in two different settings, spinal anaesthesia versus general anaesthesia. The present trial is not considered a drug study as other variables associated with type of anaesthesia are not under control of the investigators. The present trial follows guidelines of the declaration of Helsinki (version 19 October 2013). The protocol (protocol number NL54115.015.15) is approved by the Medical Ethics Committee of Máxima Medical Centre, Veldhoven, the Netherlands. The study protocol (version 1) is registered at www.trialregister.nl (NTR registration number: 5586). The present paper is written according to the Standard Protocol Items: Recommendations for Interventional Trials (SPIRIT) 2013 Statement for reporting a clinical trial protocol [16]. The SPIRIT Checklist is provided as Additional file 1.

### Participants

**Fig. 1:**
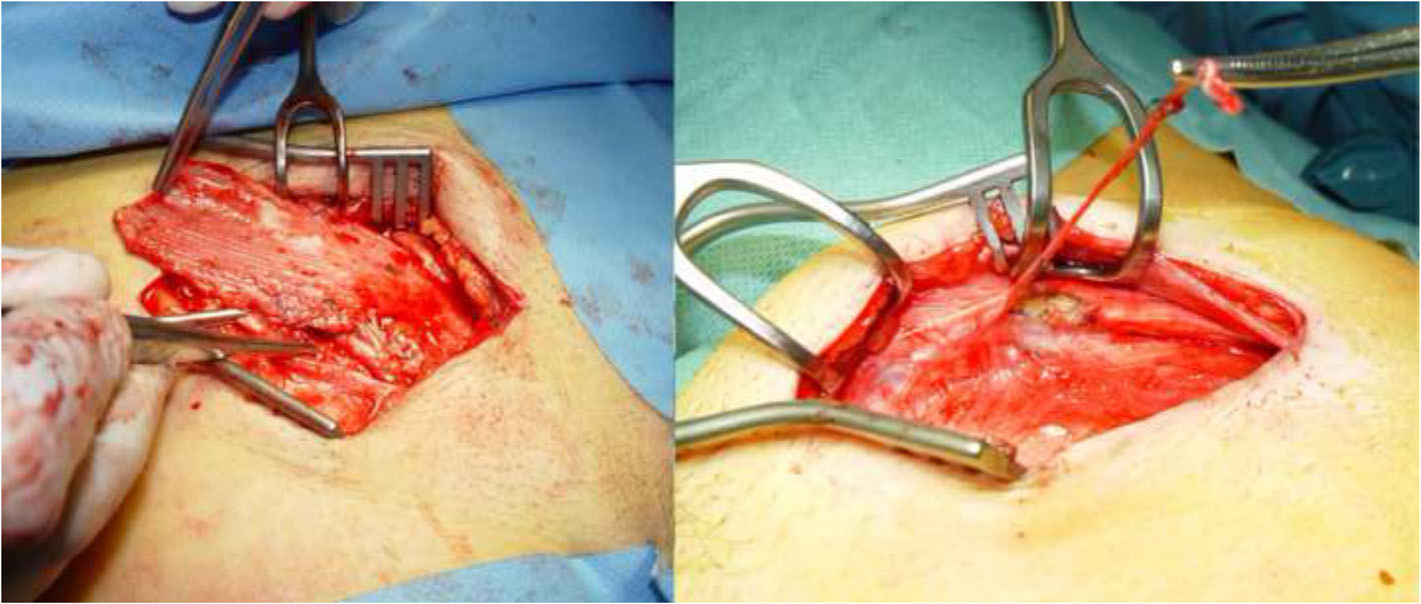
Mesh removal (*left*) and a neurectomy (*right*) in patients with inguinodynia following open inguinal hernia repair

Patient enrolment started in January 2016. Chronic inguinodynia that is unacceptable as subjectively judged by patients themselves is a prerequisite for inclusion. Only patients scheduled to undergo remedial surgery including a neurectomy and/or a meshectomy (if patients have mesh) by an open approach are considered for inclusion (Fig. 1). Patients are excluded if pain is due to intercostal neuralgia of the abdominal wall, such as abdominal cutaneous nerve entrapment syndrome (ACNES), or due to lateral femoral cutaneous nerve entrapment. Other contraindications are listed in Table 1. Cognitively impaired individuals, patients with ASA class IV or undergoing secondary or bilateral remedial surgery are also excluded.

**Table 1:** Contraindications for spinal and general anaesthesia in the present trial

Relative contraindications	Absolute contraindications
*Spinal anaesthesia*	
Deformities of the spine^a^	Refusal by patient
Severe back pain or headache	Inadequate coagulation
	Pre-existent neurological deficiency
	Infection near puncture site
	Allergy to local anaesthetic
	Inability to communication properly
*General anaesthesia*	
Expected difficulty with airway	Refusal by patient
	Allergy for (components of) medications

^a^E.g. arthritis, osteoporosis, metastasis, spinal disc herniation, scoliosis

### Interventions

The decision to perform a neurectomy and/or mesh removal depends on the surgeon’s subjective interpretation of patient history, physical examination and intraoperative findings [17]. All remedial surgeries are performed using an open approach as previously published [15]. Patients are randomised to either spinal or general anaesthesia. Hyperbaric articaine 5% is used for spinal anaesthesia, considering the relatively short duration of action. Articaine is a amide-type local anaesthetic agent that is preferentially used for short (1 h or less) surgical procedures in MMC. General anaesthesia is given to the patients following standardised procedures.

The administration of other medications during the perioperative phase (e.g. ephedrine, atropine and sedatives) is left to the discretion of the attending anaesthesiologist. Consequently, the setting of the two types of anaesthesia rather than the actions of the anaesthetic drugs are investigated. It is allowed for patients who are randomised to the spinal anaesthesia group to receive midazolam during the procedure. Midazolam has no effect on perception of pain so no drug-related sequelae on the primary outcome measure are expected. If requested, midazolam will be administered in a dose of 1 mg (if aged over 65 years) or 2 mg (if ASA class below III).

If a patient receives spinal anaesthesia but intrathecal articaine has insufficient effect, the patient will undergo surgery under general anaesthesia. Subsequently, this will be considered as a protocol violation and the particular patient will be excluded from further analysis.

### Outcomes

**Fig. 2:**
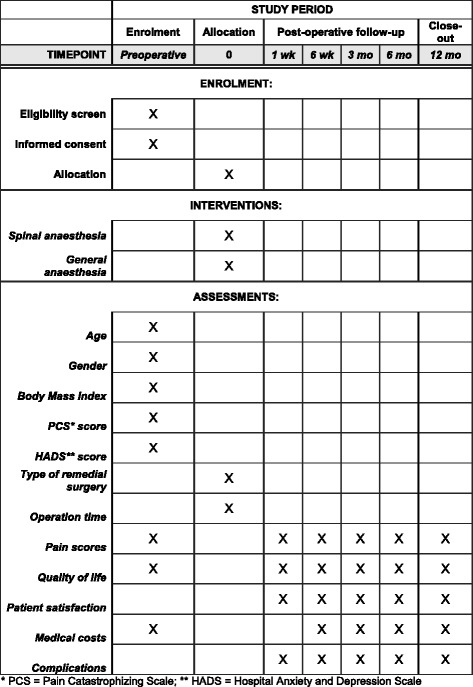
Content for the schedule of enrolment, interventions and assessments according to the SPIRIT Statement [16]

Primary outcome is the effect of type of anaesthesia on pain relief after surgery. Patients are asked to score their pain using the Numerical Rating Scale (NRS, 0, absent pain – 10, worst pain imaginable). The first pain score is obtained in the preoperative phase (*t*_0_), Fig. 2. Follow-up pain levels are determined at five time points to determine short-term (1 week, 6 weeks and 3 months postoperatively, *t*_1–_ *t*_3_) and long-term results (6 months and 12 months postoperatively, *t*_4–_ *t*_5_).

Secondary outcome measures are the effect of type of anaesthesia on quality of life and patient satisfaction. Quality of life is measured using the Short Form Health Survey-12 questionnaire (SF-12). To avoid confusion, satisfaction is also measured by a NRS-like method, using an 11-point rating scale.

Other secondary endpoints include differences in pain medication and both direct and indirect medical costs. Two different validated questionnaires, developed by the institute for Medical Technology Assessment (iMTA, Erasmus University Rotterdam, www.imta.nl) are used [18]. Complications (both surgery- and anaesthesia-related) are scored as based on the complication register of the Dutch Society for Anaesthesiologists. They are classified using the validated Clavien-Dindo classification [19]. Differences between various types of remedial surgery and specific causes of inguinodynia (primary inguinal hernia repair, primary Pfannenstiel incision, other index surgery, idiopathic inguinodynia) in relation to efficacy are also determined.

### Sample size

**Fig. 3:**
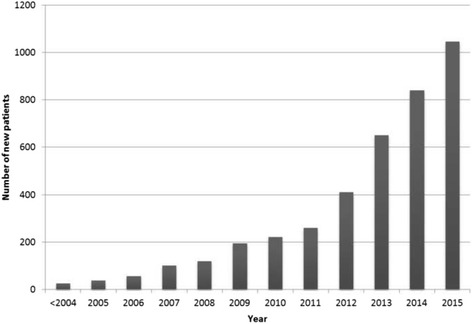
Number of unique patients analysed at SolviMáx Centre of Excellence for Chronic Abdominal Wall and Groin Pain over the years

SolviMáx was founded in 2011. An increasing number of patients with potential abdominal wall or groin pain syndromes are referred to the surgical specialists (Fig. 3). Approximately 840 unique patients were analysed in 2014, and 1045 patients in 2015. Approximately 40–45% of these undergo remedial surgery.

**Fig. 4:**
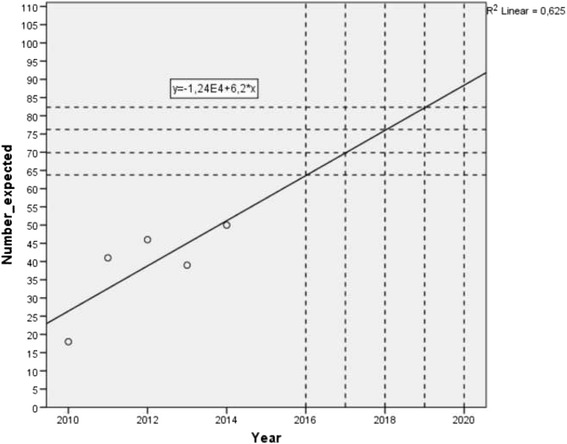
Expected inclusion in the present trial, calculated by included patients in previously performed retrospective studies [15]

Sample size was calculated by using a web-based calculator (www.openepi.com). Results from previous retrospective studies were used as criteria for eligibility were similar (Zwaans WA, van Kuijk SM, le Mair LH, van Kleef M, Scheltinga MR, Roumen RM – unpublished observations). Additional literature regarding anaesthesia on remedial surgery is not available. Previously demonstrated success rates were 77% in the spinal anaesthesia group and 58% in the general anaesthesia group (Zwaans WA, van Kuijk SM, le Mair LH, van Kleef M, Scheltinga MR, Roumen RM – unpublished observations). Based on these data, a sample size of 190 patients is calculated to demonstrate a potential effect of either type of anaesthesia on pain relief. This volume of patients is attained after three years as based on the number of included patients in previously performed studies (Fig. 4).

### Randomisation

Patients will be electronically randomised by the web-based research software Research Manager (RM, Cloud 9 Health Solutions^©^). No blocked randomisation or prestratification is performed. The decision to execute a neurectomy and/or mesh removal depends on patient history, physical examination and intraoperative findings [17]. Therefore, a prestratified randomisation is not possible for type of remedial surgery. Consequently, stratification will be executed within the analysis.

#### Sequence generation, allocation concealment and implementation

RM is used to establish an appropriate sequence generation and allocation concealment. RM generates a random allocation sequence, after which patients will be enrolled by the coordinating investigator. By using RM software, foreknowledge of (upcoming) anaesthesia assignments by the investigators is secured. Consequently, bias due to improper randomisation techniques is minimised.

### Blinding

Blinding of anaesthesiologists, surgeons and patients is not possible. Since the study is designed to investigate the setting of type of anaesthesia, where all factors associated with anaesthesia are of interest, blinding is not conditional for a robust conclusion on efficacy of either anaesthetic technique setting.

### Statistical methods

Data analysis will be performed using SPSS version 22 (SPSS Inc. ^©^) for Windows. Digital data are easily exported from RM to SPSS. The primary outcome measure is pain relief using NRS as compared to the preoperative pain levels (*t*_0_). Pain reduction is calculated by using the following formula:

$$ PAINRELIEF=\left(1-\left(\mu NRS{t}_x/\mu NRS{t}_0\right)\right)\times 100 $$

Data of the general and spinal anaesthesia group will be compared using the Student’s *t* test or the Wilcoxon signed rank test, as appropriate.

Secondary outcome measures will be compared between groups at the various postoperative follow-up points. In addition, outcomes at each evaluation time (*t*_1–_ *t*_5_) will be compared to preoperative data within groups. The Student’s *t* test or the Wilcoxon signed rank test will be used as appropriate. *P* values ≤ 0.05 are considered statistically significant. Intention-to-treat analysis will be applied on the primary outcome measure.

### Recruitment

Patients will be recruited at SolviMáx once the shared decision is taken to perform remedial surgery. During the subsequent preoperative screening, eligibility of patients for both general and spinal anaesthesia is assessed. Within 14 days of consideration, informed consent is obtained. If the patient consents, the coordinating investigator will randomise the patient as previously described. Patients are allowed to withdraw at any time point during the study.

## Discussion

General anaesthesia is still applied in the majority (60–70%) of hernia operations [20]. Spinal anaesthesia, on the other hand, is only performed in 10–20% patients undergoing inguinal hernia surgery [20], although the beneficial effects regarding postoperative pain were previously demonstrated in *primary* hernia repair [21, 22]. In addition, studies on *primary* inguinal herniorrhaphy showed that spinal anaesthesia results in shorter hospital stay, less postoperative analgesic requirements, prolonged time to first analgesic requirement, equal operation room time and equal time-to-home readiness [22–24]. Only recently, a potential beneficial effect of spinal anaesthesia on surgery for groin pain was demonstrated [15]. This is the first randomised trial comparing two routine anaesthetic modalities for remedial surgery. Results of the present trial may aid in optimising care in these patient populations.

Considering groin pain relief following remedial surgery for groin pain, spinal anaesthesia is hypothesised to be superior to general anaesthesia. The assumed beneficial effect is possibly associated with the spinal block itself diminishing transmission of nociceptive signals from the operation site toward the nervous system [21, 25]. However, as potential confounding factors are not excluded, a possible beneficial effect cannot be attributed to just one particular factor. Any potential effect should be considered as the end result of the total setting of type of anaesthesia and associated factors. These confounding factors include the intrathecal administration of anaesthetics, articaine or other drugs, patient expectations, surgical stress, patient’s state (sedated or awake) during surgery, communication with patients and various other individual psychological factors. The present study is designed to generate clinically relevant conclusions that can be directly implemented in clinical practice.

In 2004, Burney and coauthors performed a randomised trial on the effects of anaesthesia in *primary* herniorrhaphy [26]. A disappointingly low recruitment rate was observed. The authors stated that patients had conceptualised concerns regarding the anaesthetic techniques, and consequently did not accept a random assignment [26]. It is theoretically possible that patients who are more anxious about the upcoming surgical procedure prefer general over spinal anaesthesia as they do not wish to experience any ‘noise’ from within the operation room. Studies have demonstrated that anxiety is a risk factor for postoperative pain [27–29]. When these patients are prone to more intense postoperative pain and thus prefer general anaesthesia, these issues may have influenced previous outcomes. In contrast, another study showed that preferred type of anaesthesia did not contribute to success after remedial surgery (Zwaans WA, van Kuijk SM, le Mair LH, van Kleef M, Scheltinga MR, Roumen RM – unpublished observations). In SolviMáx, a standard set of intake questionnaires is obtained from all preoperative patients including a Pain Catastrophising Scale (PCS) and a Hospital Anxiety and Depression Scale (HADS) [30–32]. By analysing these scores in relation to outcome, it is possible to analyse the influence of mental status in a later phase.

The majority of studies on chronic postoperative pain lack sufficient data to draw any robust conclusions. Previously, authors have claimed that extensive preoperative data, detailed characteristics of surgical procedures and measures of acute and chronic postoperative pain (up to 1 year) are essential for a proper evaluation of effects on pain [29]. The present trial design has included all of these factors and, therefore, the results can be considered of sufficient scientific evidence. Furthermore, cost-effectiveness of both anaesthetic techniques is assessed, which is critical for clinical practice.

A potential limitation of the present study is its nonblinded design. Blinding of patients is, in our opinion, unethical and hardly practicable. Moreover, the main outcomes of the present trial are subjective. Consequently, these outcomes may be contaminated by recall bias if patients enrolled in the trial are not blinded to their treatment allocation [33].

This is the first large randomised controlled trial comparing two routinely used anaesthetic techniques in remedial surgery for groin pain. There is a definite need for evidence-based strategies to optimise the results of these types of surgery. Besides pain relief, other patient-related outcome measures are assessed to include patient’s perspectives on outcome. The first study results are expected in 2019 and will be communicated via publication.

### Trial status

Period of patient recruitment.

## Additional file

Additional file 1: SPIRIT 2013 Checklist: recommended items to address in a clinical trial protocol and related documents*. (DOCX 41 kb)

## Abbreviations

ACNES: Abdominal cutaneous nerve entrapment syndrome
ASA: American Society Anesthesiologists
HADS: Hospital Anxiety and Depression Scale
iMTA: Institute for Medical Technology Assessment
MMC: Máxima Medical Center
NRS: Numerical Rating Scale
PCS: Pain Catastrophising Scale
RM: Research Manager
SF-12: Short Form Health Survey-12 questionnaire
